# Effect of a Pay-for-Performance Program on Renal Outcomes Among Patients With Early-Stage Chronic Kidney Disease in Taiwan

**DOI:** 10.34172/ijhpm.2021.27

**Published:** 2021-04-13

**Authors:** Min-Ting Lin, Chien-Ning Hsu, Chien-Te Lee, Shou-Hsia Cheng

**Affiliations:** ^1^Institute of Health Policy and Management, College of Public Health, National Taiwan University, Taipei, Taiwan.; ^2^Department of Pharmacy, Kaohsiung Chang Gung Memorial Hospital, Kaohsiung, Taiwan.; ^3^School of Pharmacy, Kaohsiung Medical University, Kaohsiung, Taiwan.; ^4^Division of Nephrology, Department of Medicine, Kaohsiung Chang Gung Memorial Hospital, Kaohsiung, Taiwan.; ^5^College of Medicine, Chang Gung University, Taoyuan, Taiwan.; ^6^Population Health Research Center, National Taiwan University, Taipei, Taiwan.

**Keywords:** Pay-for-Performance, Chronic Kidney Disease, Cohort Study, Renal Outcome, Electronic Medical Records, Taiwan

## Abstract

**Background:** With the promising outcomes of the pre-ESRD (end-stage renal disease) pay-for-performance (P4P) program, the National Health Insurance Administration (NHIA) of Taiwan launched a P4P program for patients with early chronic kidney disease (CKD) in 2011, targeting CKD patients at stages 1, 2, and 3a. This study aimed to examine the long-term effect of the early-CKD P4P program on CKD progression.

**Methods:** We conducted a matched cohort study using electronic medical records from a large healthcare delivery system in Taiwan. The outcome of interest was CKD progression to estimated glomerular filtration rate (eGFR) <45 mL/min/1.73 m^2^ between P4P program enrolees and non-enrolees. The difference in the cumulative incidence of CKD progression between the P4P and non-P4P groups was tested using Gray’s test. We adopted a cause-specific (CS) hazard model to estimate the hazard in the P4P group as compared to non-P4P group, adjusting for age, sex, baseline renal function, and comorbidities. A subgroup analysis was further performed in CKD patients with diabetes to evaluate the interactive effects between the early-CKD P4P and diabetes P4P programs.

**Results:** The incidence per 100 person-months of disease progression was significantly lower in the P4P group than in the non-P4P group (0.44 vs. 0.69, *P*<.0001), and the CS hazard ratio (CS-HR) for P4P program enrolees compared with non-enrolees was 0.61 (95% CI: 0.58–0.64, *P*<.0001). The results of the subgroup analysis further revealed an additive effect of the diabetes P4P program on CKD progression; compared to none of both P4P enrolees, the CS-HR for CKD disease progression was 0.60 (95% CI: 0.54–0.67, *P*<.0001) for patients who were enrolled in both early-CKD P4P and diabetes P4P programs.

**Conclusion:** The present study results suggest that the early-CKD P4P program is superior to usual care to decelerate CKD progression in patients with early-stage CKD.

## Background

**Key Messages**

**Implications for policy makers**

- Clinical guideline-based pay-for-performance (P4P) programs are an applicable strategy for more active early-stage chronic kidney disease (CKD) disease management.
- While designing P4P programs, policy-makers can address the elements of continuity of care and patient adherence, which are beneficial to patient outcomes.
- There is a potential synergistic effect of more than one P4P intervention. Policy-makers may evaluate these effects from the perspective of cost-effectiveness for patients with multiple chronic diseases.

**Implications for the public**

Early-stage chronic kidney disease (CKD) earns less public awareness in the field of chronic disease management. As part of the worldwide trend to shift the focus from the treatment of late-staged kidney disease to more proactive early interventions and preventions, the National Health Insurance Administration (NHIA) in Taiwan launched the early-CKD pay-for-performance (P4P) program in 2011. This long-term study demonstrated the clinical benefits of this program in reducing and delaying patients’ kidney disease progression.

The expanding size of the population of patients with chronic kidney disease (CKD) and CKD-related morbidity and deaths have resulted in a great burden and challenges to the health systems around the world. The aging population, accompanied by an elevating prevalence of diabetes and hypertension, has further amplified the problems of CKD and end-stage renal disease (ESRD).^1-3^ The global number of patients who require maintenance dialysis for ESRD has been increasing at a rate of 7% per year,^4^ and the number of ESRD patients needing maintenance dialysis or kidney transplantation has been projected to increase from 2.618 per million population, as estimated in 2010, to 5.439 per million population in 2030.^5^ Deaths attributable to CKD are expected to rise from 12.2/100 000 in 2012 to 14/100 000 in 2030.^6,7^ Once CKD progresses to the late stage, high medical utilization and expenditures are inevitable.^4,8,9^

 Taiwan has been facing its CKD epidemic for years, with its prevalence of CKD increasing from 1.99% in 1996 to 11.9% in 2006^10^; in 2016, the incidence and prevalence rates of ESRD reached 493 and 3392 per million population, respectively, both ranking at the top in the international comparisons according to a US Renal Data Systems report.^3^ The treatment of CKD has brought a heavy financial burden to the healthcare system in Taiwan.^11^ In 2018, the cost of outpatient dialysis accounted for 8.7% of all outpatient expenditures under the National Health Insurance system.^12^

 To manage the financial and clinical burden of CKD, the health authority in Taiwan has implemented various programs to raise population awareness of the risk factors for CKD, promote organ donation, increase the number of renal transplants, establish monitoring indicators and initiate a surveillance database. In 2006, the National Health Insurance Administration (NHIA) launched a countrywide pre-ESRD pay-for-performance (P4P) program which aims to deliver adequate care for patients with proteinuria and patients with CKD at estimated glomerular filtration rate (eGFR) stage 3b (30-44.9 mL/min/1.73 m^2^), stage 4 (15-29.9 mL/min/1.73 m^2^), and stage 5 (<15 mL/min/1.73 m^2^) while not on dialysis; the effectiveness of this program in slowing the deterioration of patients’ renal function and lengthening the time to the initiation of maintenance dialysis had been demonstrated.^13-16^

 P4P, or value-based purchasing, functions by linking financial rewards to incentivize healthcare providers to deliver predefined high-quality care to their patient populations. The P4P-based payment system has set rigorous explicit indicators of clinical performance measures based on the Kidney Disease: Improving Global Outcomes international practice guidelines for renal care.^17^ Following the implementation of the pre-ESRD P4P program, the NHIA in Taiwan initiated the early-CKD P4P program in 2011 to provide active interventional care to patients at a less severe stage of renal dysfunction (eGFR ≥45 mL/min/1.73 m^2^). Early identification with active management is considered imperative to prevent CKD progression and its relevant complications through continuity of care and improved patient literacy.^18^ To date, there are insufficient effectiveness data for early CKD interventions in real-world practice settings. Therefore, this study aimed to assess whether the P4P program altered the risk of CKD progression using electronic medical records data from a large healthcare delivery system in Taiwan.

## Methods

###  Data Source

 This was a matched cohort study. Among CKD patients, we compared the long-term effect of the early-CKD P4P program between program enrolees and those receiving usual care. The study used the deidentified electronic medical records of the Chang Gung Research Database (CGRD), which includes 6 Chang Gung Memorial Hospitals located in North and South Taiwan^19^ that provide approximately 10% of the National Health Insurance-covered service volume.^20^

 Patients participating in the early-CKD P4P program were identified by the billing codes for the receipt of the early-CKD program in the CGRD between January 1, 2011 and December 31, 2017, and the extracted data included patient demographics, diagnoses, medications, medical procedures, and laboratory and examination results in the outpatient, inpatient, and emergency room settings.

###  Early-CKD P4P Program

 The Taiwan early-CKD P4P program focuses on CKD patients with eGFR ≥45 mL/min/1.73 m^2^. Healthcare providers by the medical institute can join this program voluntarily, and eligible patients are invited to the program primarily by nephrologists in the study setting. The P4P multidisciplinary care team includes physicians, nurses, dietitians and case managers who obtained sufficient professional training on kidney disease. Under the program, healthcare providers are required to provide predesignated physical examinations, urinalysis and hematology tests, and patient education in conformity with NHF-KDOQI (National Kidney Foundation – Kidney Disease Outcomes Quality Initiative) clinical guidelines^22^ to meet incentive criteria. Cardiologists and physicians specialized in metabolism are also encouraged to lead a team.^21^ Once enrolled, a patient is recommended to keep regular follow-up every 6 months by the same physician at the same medical site. The content of the guideline-based program is listed in Table S1 (see Supplementary file 1).

 There are three process-based and outcome-based financial incentives for the medical institutes (healthcare provider). The process-based performance measures are at initial enrolment (billing code: P4301C; financial reward: around 200 New Taiwan Dollars (NTD)/time), at the routine follow-up visits (billing code: P4302C; financial reward: around 200 NTD/time), and upon transferring to the pre-ESRD P4P program (billing code: P4303C; financial reward: around 200 NTD/patient). The outcome-based measure concerns patients’ improvement in the CKD stage or eGFR value and a treating physician will receive 400 NTD/patient if a patient meets the performance target (Table S2). The exchange rate between NTD and USD was ~30:1.

###  Study Cohort

**Figure 1:**
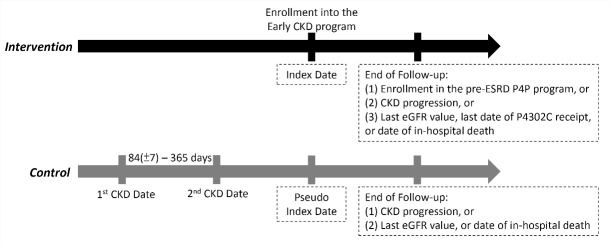
Study Design and Follow-up. Abbreviations: ESRD, end-stage renal disease; CKD, chronic kidney disease; P4P, pay-for-performance; eGFR, estimated glomerular filtration rate, calculated using the Modification of Diet in Renal Disease equation23-25: 175 x (serum creatinine) -1.154 x (Age) - 0.203 x [0.742 if female].

**Figure 2:**
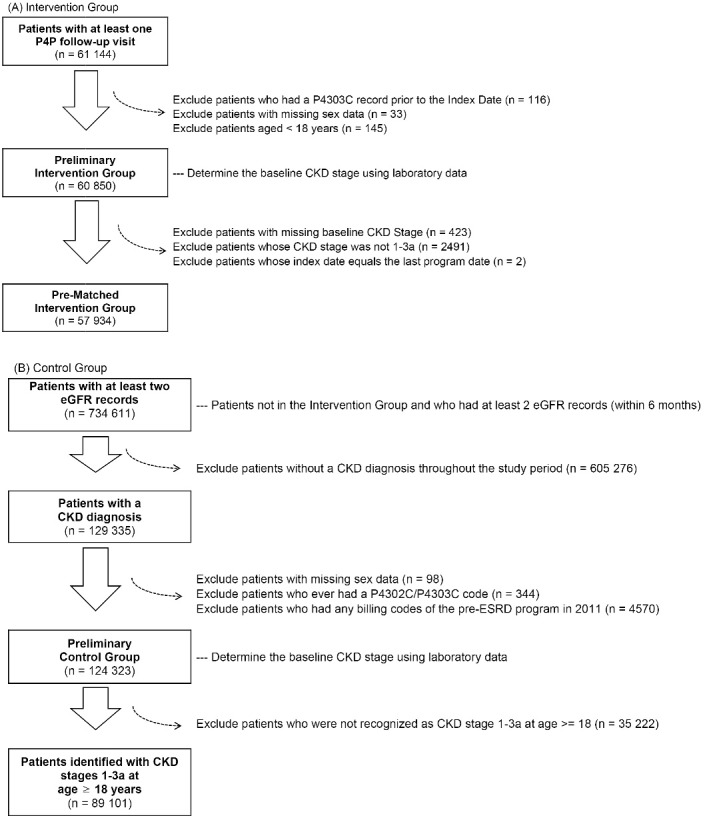
Inclusions and Exclusions of the Study Cohort. Abbreviations: P4P, pay-for-performance; ESRD, end-stage renal disease; CKD, chronic kidney disease; eGFR, estimated glomerular filtration rate; P4302C, billing code for follow-up visit in the early-CKD P4P program; P4303C, billing code for transferring to the pre-ESRD P4P program.

 The study cohort consisted of adult patients with CKD and eGFR ≥ 45 mL/min/1.73 m^2^. Patients were classified into the P4P (intervention group) and non-P4P (control group) groups based on enrolment in the early-CKD P4P program (Figure 1). To ensure the eligibility of the patients in the P4P group, we only included patients having at least one follow-up visit (P4302C). The date of an initial enrolment (P4301C) record, which was within the 3 months before the selected P4302C record, was set as the index date for individual patients in the intervention group. If no P4301C record within the previous 3 months was found, the nearest P4301C record within the previous 365 days was used instead. Baseline CKD stage at the index date was calculated using the mean eGFR value within 6 months before the index date. Figure 2A provides detailed information about the inclusion and exclusion criteria.

 From the patients receiving usual care in the study setting (control group), we first identified those who had at least two eGFR values within an interval of 6 months to comprise the candidates for the control group. Second, a patient with the CKD diagnosis based on International Classification of Disease – 9 or 10 – Clinical Modification (ICD-9/10-CM) codes was defined as having at least two claims of CKD in outpatient or inpatient visits with an interval of 84 (±7) to 365 days following the NHF-KDOQI guidelines.^22^ We adopted the ICD-9-CM codes for CKD in the CGRD before 2016^26^ and ICD-10-CM codes for CKD in the CGRD between 2016 and 2017. Patients were excluded if they (1) had only one accidental eGFR value, (2) lacked the 6-month interval between eGFR data (to be consistent with patients in the intervention group), or (3) ever had a P4302C or P4303C code or had any billing codes for the pre-ESRD P4P program in 2011.

 In the control group, if a patient had more than one CKD stage (calculated using eGFR values) documented during the study period, only one stage was randomly selected for later matching purposes, and the date of the selected CKD stage served as a temporary index date for retrieving the patient’s baseline information. Finally, to avoid attrition bias, patients whose follow-up duration was less than 3 months were excluded (Figure 2B).

 To minimize selection bias, a 1:1 exact matching strategy was employed, according to patient age (by 5 years), sex, number of baseline comorbidities according to the Charlson Comorbidity Index (CCI),^27^ baseline eGFR value and the year and quarter (a 3-month interval) of the eGFR value, and the duration of follow-up (number of 6-month intervals) for potential bias of missing data. In the matched cohort, patients of the control group were assigned with a pseudo index date of their counterparts (matched individual in the intervention group). Baseline eGFR was determined by the mean values of at least two eGFRs within 6 months before the index date (stage 1: eGFR ≥90 mL/min/1.73 m^2^, stage 2: eGFR = 60-89 mL/min/1.73 m^2^, and stage 3a: eGFR = 45-59 mL/min/1.73 m^2^). The individual comorbid disease in the CCI was identified as at least one disease-specific ICD-9/10-CM code that appeared in the outpatient, inpatient, or emergency room setting within the past 365 days before the index date.

###  Outcomes

 The primary outcome was CKD progression, recognized as eGFR < 45 mL/min/1.73 m^2^ that occurred in two consecutive quarters apart between 84-190 days, and the earlier date of eGFR <45 was defined as the first event of interest. In the intervention group, having a P4303C code, which indicated enrolment in the pre-ESRD P4P program, was an alternative endpoint criterion, and the event date for CKD progression was the date of eGFR <45 mL/min/1.73 m^2^ or the P4303C code presented, whichever came first. Patients who did not experience the outcome of interest were censored at date of the last available eGFR value, the last date of P4302C receipt, or the date of in-hospital mortality, whichever occurred later. Death events were ascertained using hospitalization discharge records and validated by the absence of any healthcare services encounters afterward.

 The study followed patients from index date to the first event of eGFR <45 mL/min/1.73 m^2^, death or censored date. Program interruption was defined as two consecutive documented P4P billing codes that were separated by more than 365 days. If a patient’s last date of P4P receipt appeared to be the only record after an interruption, that record would not be adopted as the censoring date.

###  Statistical Analysis

 Covariates included patient age, sex, baseline eGFR level, and comorbidities based on CCI disease conditions.^27,28^ We calculated the eGFR level by the Modification of Diet in Renal Disease equation^23-25^: 175 × (serum creatinine) -1.154 × (Age) - 0.203 × [0.742 if female], where the laboratory data were collected within six months before the index date. Enrolment in the diabetes P4P program during the baseline 12-month period was defined as at least one billing code that denoted the follow-up visits in the diabetes P4P program (P1408C, P1409C, P1410C, P1411C).

 Student’s *t *test was used for continuous data, and the χ^2^ test for categorical data for comparing the variables between groups. The cumulative incidence of CKD progression was compared between the two groups with Gray’s test to avoid overestimated probability of the CKD progression event. In-hospital mortality was considered a competing event in cause-specific (CS) hazard regression model to estimate the adjusted hazard ratio (HR) with a 95% confidence interval (CI) for the intervention, adjusting for age, sex, baseline eGFR levels, diabetes P4P program and CCI score.

 A subgroup analysis for patients with diabetes was carried out to assess the heterogeneity of the early-CKD P4P program effects in different subgroups of patient and the interactive effects between the early-CKD P4P and the diabetes P4P programs (the latter was implemented in 2001). A two-sided test with a *P* value of <.0500 was considered statistically significant. All statistical analyses were performed using SAS version 9.4 (SAS Institute, Cary, NC, USA).

## Results

###  Patient Characteristics

Figures 2A and 2B show the patient selection process. The number of patients in the full study cohort from 2011 to 2017 was 57 934, and the different categories denote the duration between each patient’s first enrolment and end of follow-up date (Figure S1). Based on the definition of program interruption, 95% of the enrolled patients did not experience any interruption, suggesting a good adherence rate for the program. A total of 2719 of the patients had one interruption, 126 patients had two interruptions, and two patients had three interruptions.

 The baseline patient characteristics before and after matching are summarized in Table 1. Before matching, patients in the control group tended to be younger, had a greater proportion of CKD stage 1 disease than stage 3a disease, and had fewer comorbid diseases. The matched cohort consisted of 45 770 patients, with 59.4% of the patients being male. The mean age of this patient cohort was 64.6 (±12.1) years, most (97.1%) of the patients were at stage 2 or 3a CKD, and more than 80% of the patients had 0-2 CCI disease categories. A greater proportion (17.9% vs. 6.5%) of patients in the intervention group was also enrolled in the diabetes P4P program at baseline.

**Table 1:** Baseline Characteristics of Patients Before and After Matching

**Characteristics**	**Before Matching**					**After Matching**				
	**Control Group ** **(N = 81 816)**		**Intervention Group ** **(N = 57 934)**		***P*** ** Value**	**Control Group ** **(N = 22 885)**		**Intervention Group ** **(N = 22 885)**		***P*** ** Value**
**Matching Keys**										
**Gender**					<.0001					1.0000
Male	45695	(55.9%)	32956	(56.9%)		13594	(59.4%)	13594	(59.4%)	
Female	36121	(44.1%)	24978	(43.1%)		9291	(40.6%)	9291	(40.6%)	
**Age, years**					<.0001					.8988
Mean ± SD	61.0 ± 14.5		65.2 ± 13.2			64.6 ± 12.1		64.6 ± 12.1		
Median	61.3		65.7			64.8		64.8		
Min-Max	18-103.0		18-103.2			18-98.2		18-98.4		
**CKD stage**					<.0001					1.0000
Stage 1	25980	(31.8%)	1168	(2.0%)		671	(2.9%)	671	(2.9%)	
Stage 2	33908	(41.4%)	30 426	(52.5%)		12530	(54.8%)	12530	(54.8%)	
Stage 3a	21928	(26.8%)	26 340	(45.5%)		9684	(42.3%)	9684	(42.3%)	
**Number of baseline CCI disease category **					<.0001					1.0000
0	23117	(28.3%)	9105	(15.7%)		4888	(21.4%)	4888	(21.4%)	
1	29543	(36.1%)	14 369	(24.8%)		7723	(33.7%)	7723	(33.7%)	
2	18609	(22.7%)	19 224	(33.2%)		6926	(30.3%)	6926	(30.3%)	
3	7246	(8.9%)	9651	(16.7%)		2430	(10.6%)	2430	(10.6%)	
4+	3301	(4.0%)	5585	(9.6%)		918	(4.0%)	918	(4.0%)	
**Non-matching Keys**										
**Baseline CCI score**										.3241
Mean ± SD						1.9 ± 1.7		1.9 ± 1.9		
Median						2.0		2.0		
Min-Max						0-16		0-16		
**Baseline diabetes P4P enrolment **										<.0001
Yes						1480	(6.5%)	4107	(17.9%)	
No						21405	(93.5%)	18778	(82.1%)	

Abbreviations: CKD, chronic kidney disease; CCI, Charlson comorbidity index; P4P, pay-for-performance; SD, standard deviation. Stage 1: eGFR ≥90 mL/min/1.73 m^2^; stage 2: eGFR 60-89.9 mL/min/1.73 m^2^; stage 3a: eGFR 45-59.9 mL/min/1.73 m^2^.

###  CKD Progression

 The overall mean follow-up was 27.9 (±19.0) months, and 30.5 (±19.1) months for patients in the intervention group, 25.3 (±18.6) months for the control group. Over the study period, the decline in the mean eGFR per month was significantly smaller in the intervention group than the control group (-0.22 [±1.13] mL/min/1.73 m^2^ versus -0.30 [±2.66] mL/min/1.73 m^2^, *P* <.0001). Table 2 summarizes the cumulative eGFR <45 mL/min/1.73 m^2^ or transfer to the pre-ESRD intervention program events. During 5 years of follow-up, the incidence rate of CKD progression was significantly lower in the intervention group than in the control group (rate ratio = 0.64, 95% CI = 0.61–0.67, *P* <.0001). A similar trend could be found in patients at stage 2 and stage 3a CKD. There were approximately 5% of patients who remained in both study groups after 60 months of follow-up, thus time-to-event estimates were performed at 60 months.

**Table 2:** Incident CKD Progression Events, Overall and by Baseline CKD Stages Over 5 Years

	**Control Group**	**Intervention Group**	**Rate Ratio**	
			**(95% CI)**	***P*** ** Value**
Overall				
N	22885	22885		
No. of events	3913	2998		
Rate per 100 patient-months	0.69	0.44	0.64 (0.61–0.67)	<.0001
Stage 1				
N	671	671		
No. of events	4	6		
Rate per 100 patient-months	0.02	0.03	1.35 (0.38–4.79)	.6413
Stage 2				
N	12530	12530		
No. of events	712	587		
Rate per 100 patient-months	0.20	0.15	0.73 (0.66–0.82)	<.0001
Stage 3a				
N	9684	9684		
No. of events	3197	2405		
Rate per 100 patient-months	1.60	0.90	0.56 (0.54–0.60)	<.0001

Abbreviations: CKD, chronic kidney disease; eGFR, estimated glomerular filtration rate, calculated using the Modification of Diet in Renal Disease equation (175 × (Scr) -1.154 × (Age)-0.203 × (0.742 if female)).

**Figure 3:**
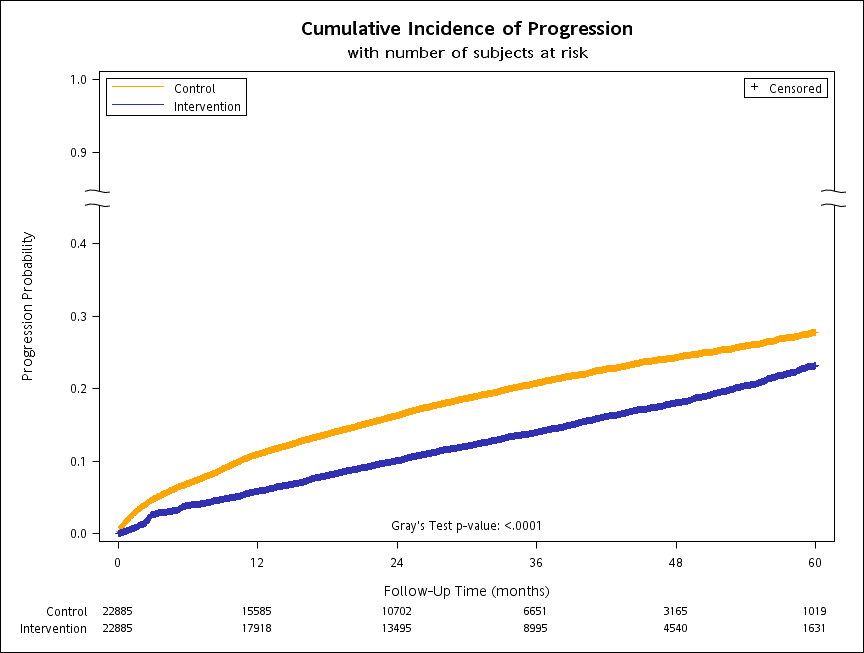
Cumulative Incidence of CKD Progression Over 5 Years. Abbreviation: CKD, chronic kidney disease.

 The cumulative incidence curves are shown in Figure 3, with the result of Gray’s test suggesting significantly different incidence profiles between the groups. The prevention of CKD progression in the intervention group remained after adjusting for the patients’ demographics and CCI disease conditions; that is, patients included in the P4P group demonstrated a 39% reduced risk of disease progression after adjustment (CS-HR: 0.61, 95% CI: 0.58–0.64, *P* <.0001) (Table 3). Patients at stage 3a (CS-HR: 6.46, 95% CI: 6.07–6.88) or with a higher CCI score (CS-HR: 1.10, 95% CI: 1.09–1.12) had a significantly increased risk of CKD progression. The cumulative incidence curves were further stratified by CKD stage (Figures S2A-S2B).

**Table 3:** Factors Associated with CKD Progression

**Parameters**	**CS-HR (95% CI)**	***P*** ** Value**
Early CKD program (vs. control)	0.61 (0.58–0.64)	<.0001
Age	1.02 (1.02–1.02)	<.0001
Male (vs. female)	0.86 (0.82–0.90)	<.0001
Baseline CKD stage		
eGFR 45-59.9 (vs. ≥ 60 mL/min/1.73 m^2^)	6.46 (6.07–6.88)	<.0001
CCI score	1.10 (1.09–1.12)	<.0001
Diabetes P4P program	0.98 (0.90–1.06)	.5680

Abbreviations: CKD, chronic kidney disease; CCI, Charlson comorbidity index; CS-HR, cause-specific hazard ratio; P4P, pay-for-performance; eGFR, estimated glomerular filtration rate.

###  Subgroup Analysis

 The study results revealed an additive effect of the diabetes P4P program on CKD disease progression among early-CKD patients with diabetes at enrolment (n = 23 071). In the early-CKD intervention group, more than 30% diabetes patients had ≥1 comorbid conditions and 31.1% of them also participated in the diabetes P4P program (Table S3). Using the multivariable CS hazard regression model, we found that compared with non-P4P enrolees (neither the early-CKD program nor DM program), those who participated in the diabetes P4P program had a 21% reduced risk of CKD disease progression (CS-HR: 0.79, 95% CI: 0.68–0.92, *P* =.0024), and the CS-HR was 0.65 (95% CI: 0.60–0.70, *P* <.0001) and 0.60 (95% CI: 0.54–0.67, *P* <.0001), respectively, for patients who were enrolled in the early-CKD program and patients who were enrolled in both programs (Table 4). The effects of other covariates are consistent with those reported in the main model (Table 3). If the interaction term (both early-CKD and diabetes P4P programs) was considered in the primary model, the CS-HR for the interaction term was 1.17 (95% CI: 0.98–1.41, *P* =.0891).

**Table 4:** Factors Associated with CKD Progression in CKD Patients Comorbid with Diabetes

**Parameters**	**CS-HR (95% CI)** ^a^	***P*** ** Value**
(Reference: none of both P4Ps)	-	-
Diabetes P4P only	0.79 (0.68–0.92)	.0024
Early-CKD P4P only	0.65 (0.60–0.70)	<.0001
Diabetes and Early-CKD P4P	0.60 (0.54–0.67)	<.0001

Abbreviations: CKD, chronic kidney disease; CS-HR, cause-specific hazard ratio; P4P, pay-for-performance. ^a^The HRs were adjusted for age, sex, CKD stage, and CCI score.

## Discussion

 This study is one of few large cohort studies to evaluate the effectiveness of early intervention in the CKD patient population under a clinical guideline-based P4P program. The study results suggested the effectiveness of the early-CKD P4P program in reducing the risk of CKD progression among patients with an eGFR ≥45 mL/min/1.73 m^2^ compared to the usual care. Over 6 years of study follow-up, CKD patients with diabetes were found to benefit from the intervention of the early-CKD P4P program, and the benefit was greater for patients who were also enrolled in the diabetes P4P program.

 Possible explanations for the positive outcome of the P4P program on CKD progression are as follows. First, higher probability of completing regular follow-up visits among the enrolled early-CKD patients facilitates better monitoring and control of disease progression; second, in-depth health education for the enrolled patients may have increased the ability and willingness of the patients for engagement and self-management; third, the P4P program may have increased the mutual trust between the participating physicians and the enrolled patients which may lead to better adherence to guideline recommendations.

 There has been a worldwide trend to shift the focus from the treatment of ESRD to more proactive primary and secondary prevention strategies while improving the quality of CKD care.^1,29-32^ Bello et al^33^ pointed out several obstacles to effective care for non-dialysis-dependent CKD patients, including the absence of a disease surveillance mechanism and lack of a coordinated caring strategy. The present study results therefore shed light on the benefits of the P4P payment mechanism, which might incentivize coordinated and high-quality care to refine the management of early-stage chronic disease. Some studies examined the P4P program from the perspective of incentive design.^34-36^ CKD-related indicators, such as controlled blood pressure was targeted in the P4P system in the UK Quality and Outcomes Framework (QOF) in 2006. Karunaratne et al conducted a 6-year prospective cohort study to the impact of P4P system pre- and post-QOF periods (2 and 4 years, respectively) and found that the proportion of patients who reached the BP target improved from 41.5% to 50.0% and increased uses of anti-hypertensive prescriptions in CKD patients at stage 3-5 at enrolment.^34^ Hsieh et al applied population-based claims data in Taiwan and found that on the basis of process-based incentives, healthcare providers did respond to the additional outcome measures (hemoglobin A1c level and lipid profiles). Compared with the time period without outcome-based incentives, the implementation of these incentives reflected a significant decrease in hemoglobin A1c level (-1.97 vs. -5.72 % change from baseline) and LDL cholesterol (-1.87 vs. -6.10 % change from baseline)^36^ in patients with diabetes.

 Instead of examining the P4P program from the perspective of financial incentives, the present study, importantly, addressed the continuity of care resulting from the P4P program. In the study cohort, the patients’ mean duration of enrolment in the early-CKD P4P program was 29.2 (+18.9) months (results not shown), and 95% of patients did not experience any program interruption, suggesting good adherence to the program. In other words, a patient who can adhere to the program for at least 2.5 years is expected to benefit from the P4P program, as demonstrated in this study. We also conducted a sensitivity analysis by excluding the 5% of patients with program interruption from the analysis and found results consistent with the main findings (adjusted HR: 0.64, 95% CI: 0.61–0.67, *P* <.0001, results not shown).

 Lin et al extracted claims data from a multicenter cohort in southern Taiwan and pointed out that for patients with CKD stages 3b-5, adherence to the pre-ESRD P4P program was poor.^14^ Yen et al evaluated the continuity of care of the diabetes P4P program in Taiwan and found that 44.3% of P4P program enrolees failed to complete their first annual diabetes evaluation, although the P4P program did improve continuing care.^37^ A time-series analysis from the UK also showed a reduced continuity of care following the initiation of the P4P mechanism of the QOF.^38^ These inconsistent findings imply varied long-term cost-effectiveness of P4P programs among patients with different disease severities, which is worth examining in future studies.

 The current study also showed several risk factors associated with CKD progression, including older age, higher disease stage, and more severe baseline comorbidities. The study findings also echoed the interactive effects of the early-CKD P4P and diabetes P4P programs, as indicated by the Liao et al^39^ study. In Taiwan, there has been an increasing number of ESRD patients with diabetic nephropathy as their primary renal disease,^40^ and the proportion of newly diagnosed dialysis patients with comorbid diabetes increased from 34.6% in 2000 to 46.1% in 2016.^41^ In light of these facts, the spill over effect of a single P4P intervention or the synergistic effect of more than one P4P intervention make an important contribution in the management of chronic disease.

 Barriers associated with P4P participation included the amount of the incentives, the recipient of the incentives, and perceived risk of not receiving the rewards.^42,43^ Further research is warrant to explore causes of not participating in the early-CKD P4P program from the perspectives of healthcare providers, administrators and patients.

 This study was prone to several limitations that merit considerations when interpreting the study results. Although the CGRD included early-CKD patients from broad geographic areas of Taiwan, there were numerous challenges when applying real-world data derived from routine care settings. First, it was possible that patients participated in the P4P program for early-CKD or diabetes outside of the study setting. The point estimate of the treatment effect could be biased by exposure misclassification. Nevertheless, as the NHIA conducts continuous monitoring of the P4P programs on a monthly basis to prevent duplicated payments, it is unlikely that a patient persistently joined a specific P4P program in different hospitals. Missing data or lack of care continuity within the same medical intuition could lead to underestimated event rate, especially in the usual care group.

 Second, the impact of acute kidney injury (AKI) on early-CKD P4P was not specified in the analyses. For some patients who suffered from an acute decline in renal function, the measure of quarter mean of eGFR was unable to determine renal function at acute or recovery phases from AKI. It is important to note that dialysis – or hospitalization – requiring AKI is associated with long-term CKD progression and warrants future research.^44^ Third, administrator- and physician-related factors might have contributed to a selection bias that was not controlled by matching,^45^ and residual confounding may have existed as factors such as patient loyalty and different practices among different specialties of physicians were not measured. Lastly, the sustainability of the P4P program, treatment protocols, and clinical practices in the study setting might not be generalizable to other healthcare institutions.

## Conclusion

 In conclusion, the early-CKD P4P program in Taiwan showed long-term clinical benefits in patients with early-stage CKD. The empirical findings demonstrated reduced and delayed disease progression among P4P program enrolees, suggesting that P4P is an applicable strategy for more active early-stage CKD disease management. Future studies are warranted to take healthcare costs into account and to analyse the cost-effectiveness of the program from both the societal and healthcare system perspectives.

## Acknowledgements

 The authors thank Mrs. Chun-Hua Liao and Ying-Jen Hsu from the Department of Management of Information Systems, Chang Gung Memorial Foundation, for their technical assistance with the retrieval of the raw data from the electronic health records. The authors are grateful to Ying-Chun Liao, RN in the Division of Nephrology, Kaohsiung Chang Gung Memorial Hospital for intellectual discussions that assisted the methods employed in the study.

## Ethical issues

 The study proposal was approved by the Institutional Review Board (IRB) of the Chang Gung Medical Foundation (CGMF) in Taipei, Taiwan (permit number: 201800315B0C502). Individual patient consent was waived as the CGRD is an anonymized generic electronic research database. The study conduct was compliant with the guidelines and regulations of the IRB of the CGMF.

## Competing interests

 Authors declare that they have no competing interests.

## Authors’ contributions

 Conception and design: MTL, CNH, and SHC; Acquisition of data and analysis: CNH and MTL; Interpretation of data: All authors; Manuscript writing and critical revisions for important intellectual content: All authors; Approval for the final version to be submitted and any revised versions: All authors.

## Funding

 This work was supported by Kaohsiung Chang Gung Memorial Hospital (CMRPG8H0961). The funder had no role in the study design, data collection and analysis, decision to publish, or preparation of the manuscript.

## Supplementary files

Supplementary file 1 contains Tables S1-S3 and Figures S1-S2.
