# Supplementary material for: Effect of a Pay-for-Performance Program on Renal Outcomes Among Patients With Early-Stage Chronic Kidney Disease in Taiwan
Source: Int J Health Policy Manag. 2021 Apr 13;11(8):1307–15. doi: 10.34172/ijhpm.2021.27 (PMC9808322; doi:10.34172/ijhpm.2021.27)
Supplement: Supplementary file 1 — contains Tables S1-S3 and Figures S1-S2. [file ijhpm-11-1307-s001.pdf]

Article title: Effect of a Pay-for-Performance Program on Renal Outcomes Among Patients With Early-Stage Chronic Kidney Disease in Taiwan

Journal name: International Journal of Health Policy and Management (IJHPM)

Authors' information: Min-Ting Lin[1], Chien-Ning Hsu[2,3], Chien-Te Lee[4,5], Shou-Hsia Cheng[1,6]*

[1]Institute of Health Policy and Management, College of Public Health, National Taiwan University, Taipei, Taiwan.

[2]Department of Pharmacy, Kaohsiung Chang Gung Memorial Hospital, Kaohsiung, Taiwan.

[3]School of Pharmacy, Kaohsiung Medical University, Kaohsiung, Taiwan.

[4]Division of Nephrology, Department of Medicine, Kaohsiung Chang Gung Memorial Hospital, Kaohsiung, Taiwan.

[5]College of Medicine, Chang Gung University, Taoyuan, Taiwan.

[6]Population Health Research Center, National Taiwan University, Taipei, Taiwan.

(*corresponding author: shcheng@ntu.edu.tw)

Supplementary file 1

Table S1. Contents of the Guideline-based Early-CKD Program

1. Screening of CKD patients
   - eg. routine examination of urine protein-to-creatinine ratio (U_{PCR}) or urine albumin-to-creatinine ratio (U_{ACR})
2. Guidelines for the management of CKD patients comorbid with hypertension
   - eg. prescription of angiotensin-converting-enzyme inhibitors (ACEI) or angiotensin receptor blockers (ARB)
3. Guidelines for the management of CKD patients comorbid with diabetes
   - eg. attainment of blood glucose and HbA1c target values
4. Guidelines for the management of CKD patients comorbid with hyperlipidemia
   - eg. routine examination of fasting lipid profile, including total cholesterol, triglyceride, LDL-C and HDL-C
5. Lifestyle modification recommendations
   - eg. smoking cessation, body weight reduction
6. Medication use recommendations
   - eg. avoidance of nonsteroidal anti-inflammatory drugs (NSAIDs) and aminoglycoside

Table S2. Outcome-based Incentive in the Early-CKD Program

If a patient meets any of the below two criteria, the treating physician will receive financial reward (around 400 NTD for each patient).

Criterion 1: An improvement in the CKD stage (eg. from stage 2 at enrolment to stage 1)

Criterion 2: An improvement in the eGFR value, with an achievement in at least two of the below indicators:

- Blood pressure control: an improvement from ≥ 140/90 mmHg at enrolment to < 130/80 mmHg
- HbA1c control in patients with diabetes: an improvement from ≥ 7.0% at enrolment to < 7.0%
- LDL control: an improvement from ≥ 130 mg/dl at enrolment to < 130 mg/dl
- Smoking cessation for at least 6 months

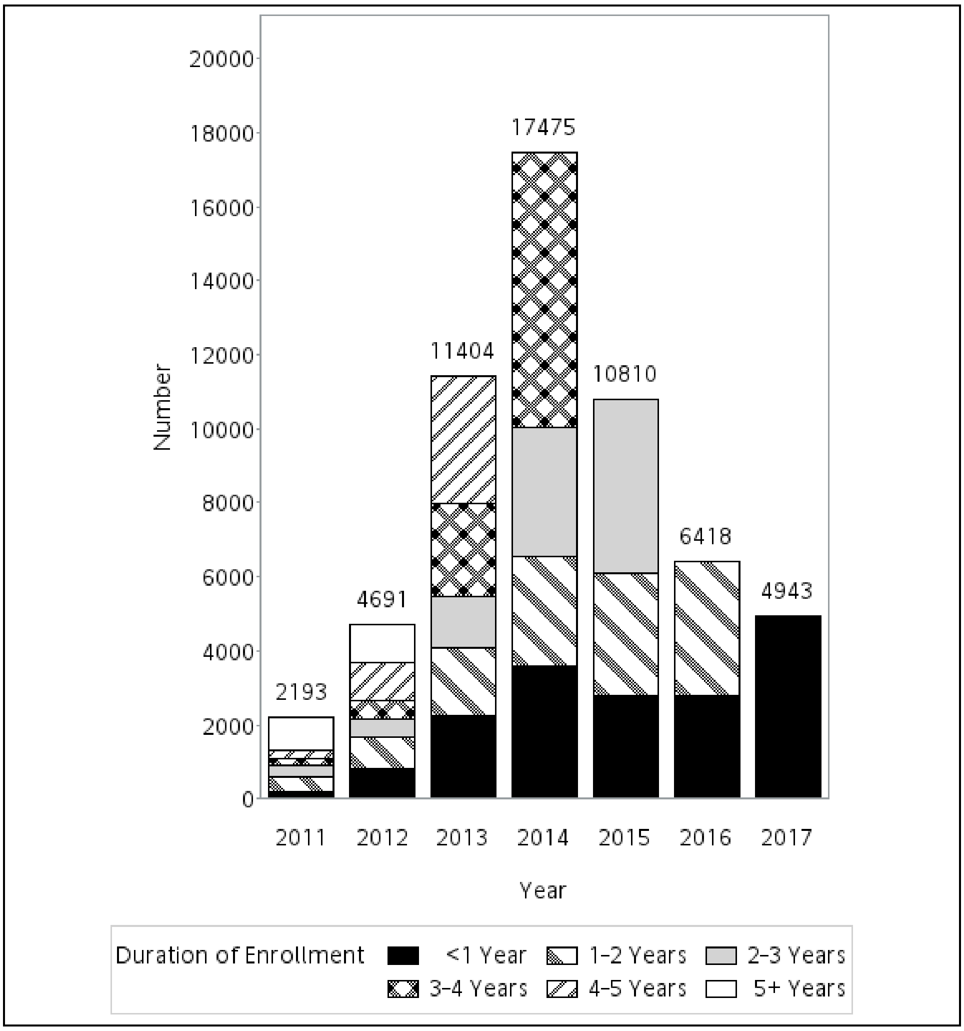

Figure S1. Number of Patients Newly-enrolled in the Early-CKD Program, Categorized by Duration of Enrolment.

(A)Stages 1-2 (eGFR ≥ 60 ml/min/1.73 m^2)

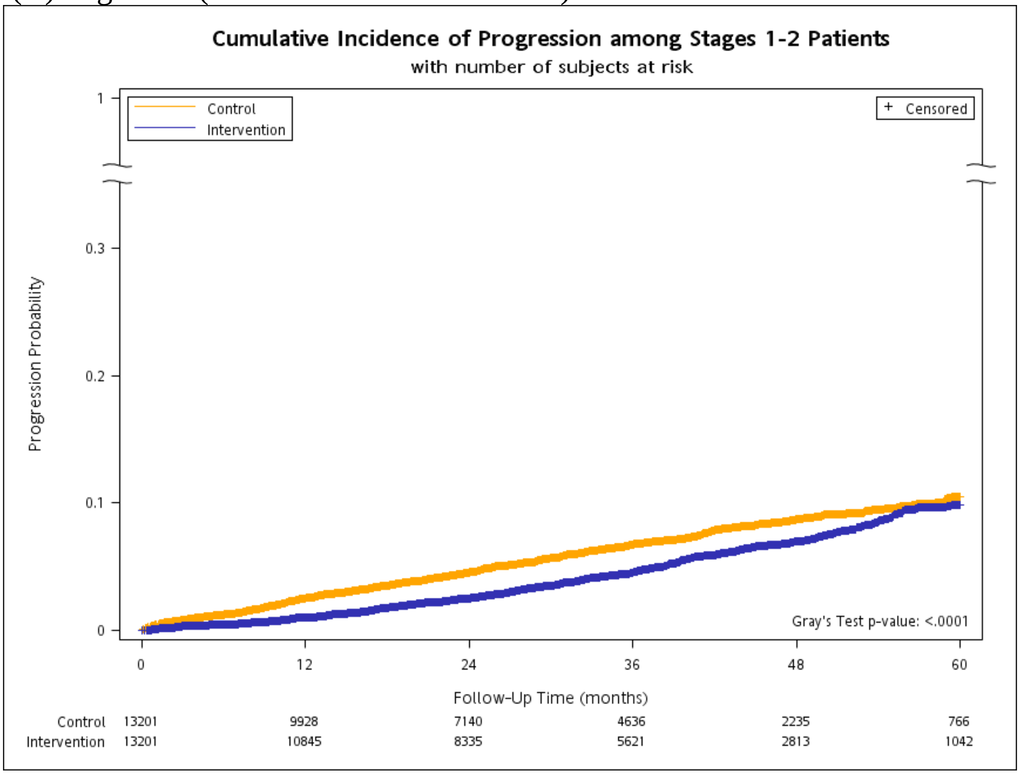

(B)Stage 3a (eGFR = 45-59.9 ml/min/1.73 m^2)

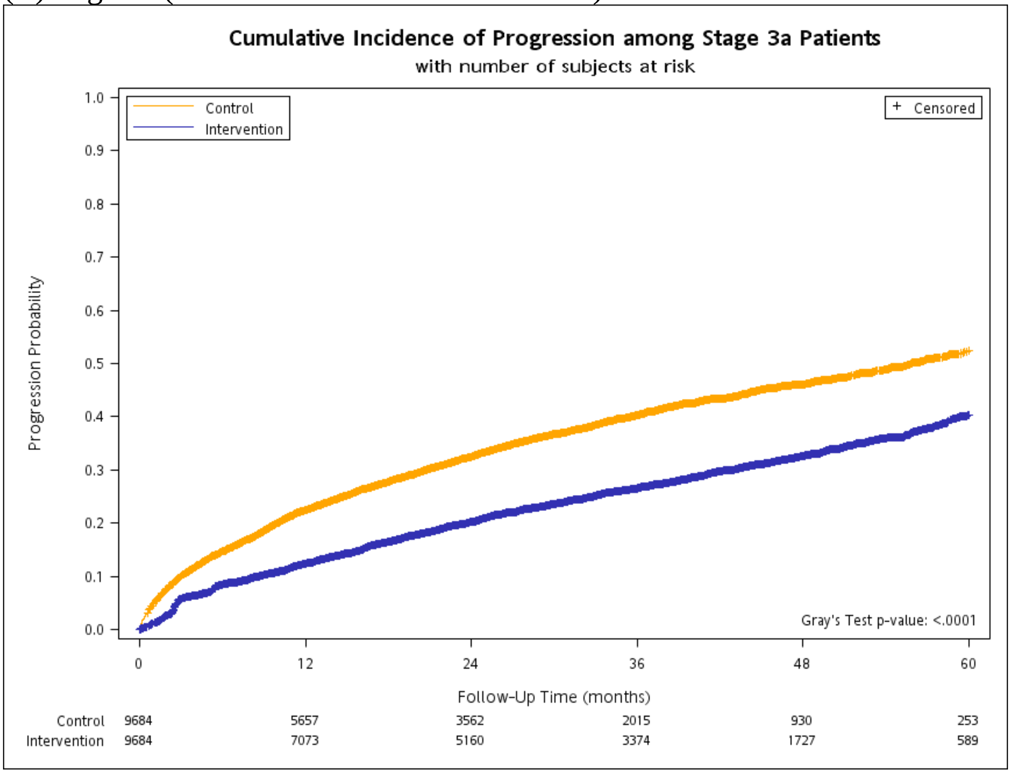

Figure S2. Cumulative Incidence of Progression by Baseline CKD Stage.

Table S3. Characteristics of CKD Patients with Diabetes at Baseline

Characteristics	Control Group (N =9,963)		Intervention Group (N =13,108)		p-value
**Male**	5677	(57.0%)	7504	(57.2%)	0.6852
**Age, years**					0.4389
Mean + SD	64.7 ± 11.0		64.6 ± 11.1		
**eGFR,** ml/min/1.73 m^2					<0.0001
Mean + SD	70.0 ± 17.5		74.9 ± 23.2		
**CKD Stage**					<0.0001
1 (eGFR ≥ 90)	374	(3.8%)	358	(2.7%)	
2 (eGFR 60-89.9)	5945	(59.7%)	8232	(62.8%)	
3a (eGFR 45-59.9)	3644	(36.6%)	4518	(34.5%)	
**Baseline CCI Score***					
Mean + SD	1.0 ± 1.5		0.6 ± 1.2		<0.0001
0	4484	(45.0%)	8534	(65.1%)	<0.0001
1-2	4499	(45.2%)	3933	(30.0%)	
>3	980	(9.8%)	641	(4.9%)	
**Baseline Diabetes P4P Enrolment**	1467	(14.7%)	4083	(31.1%)	<0.0001

*Diabetes was not included in the calculation
